# Identification of epidermal growth factor receptor and its inhibitory microRNA141 as novel targets of Krüppel-like factor 8 in breast cancer

**DOI:** 10.18632/oncotarget.4077

**Published:** 2015-05-27

**Authors:** Tianshu Li, Heng Lu, Debarati Mukherjee, Satadru K. Lahiri, Chao Shen, Lin Yu, Jihe Zhao

**Affiliations:** ^1^ Burnett School of Biomedical Sciences, University of Central Florida College of Medicine, Orlando, FL, USA; ^2^ Department of Cancer Biology, Lerner Research Institute, Cleveland Clinic, Cleveland, OH, USA; ^3^ College of Life Sciences, Wuhan University, Wuhan, China

**Keywords:** KLF8, EGFR, microRNA141, invasion and metastasis, breast cancer

## Abstract

Krüppel-like factor 8 (KLF8) is a dual transcriptional factor critical for breast cancer progression. Epidermal growth factor receptor (EGFR) is frequently overexpressed in aggressive such as triple-negative breast cancer and associated with poor clinical outcomes. Here we report a novel KLF8-EGFR signaling axis in breast cancer. We identified a highly correlated co-overexpression between KLF8 and EGFR in invasive breast cancer cells and patient tumor samples. Overexpression of KLF8 in the non-tumorigenic MCF-10A cells induced the expression of EGFR, whereas knockdown of KLF8 from the MDA-MB-231 cells decreased it. Promoter activation and binding assays indicated that KLF8 promotes the EGFR expression by directly binding its gene promoter. We also revealed that KLF8 directly represses the promoter of miR141 and miR141 targets the 3′-untranslational region of EGFR transcript to inhibit EGFR translation. Treatment with the EGFR inhibitor AG1478 or overexpression of miR141 blocked the activity of ERK downstream of EGFR and inhibited KLF8-depndent cell invasiveness, proliferation and viability in cell culture and invasive growth and lung metastasis in nude mice. Conversely, overexpression of an inhibitory sponge of miR141 led to the opposite phenotypes. Taken together, these findings demonstrate a novel KLF8 to miR141/EGFR signaling pathway potentially crucial for breast cancer malignancy.

## INTRODUCTION

Breast cancer is one of the leading causes of cancer death among women with the invasive growth and metastasis being the major cause of patient death. Thorough understanding of the underlying mechanisms has been a main focus of breast cancer research for developing more effective therapies to improve patient survival.

Krüppel-like factor 8 (KLF8) is a dual transcription factor [1–9] that is overexpressed in various types of human cancer including breast cancer [2, 8, 9, 11]. It plays a crucial role in promoting the cell cycle progression [10–12], transformation [3, 14], epithelial to mesenchymal transition (EMT) [1, 7, 13, 14] and DNA damage repair [2]. Although much progress has been made in its transcriptional and post-translational roles and regulation as well as its nuclear localization [1, 2, 4, 8], the molecular and signaling mechanisms by which KLF8 promotes human breast cancer progression remain largely elusive.

Epidermal growth factor receptor (EGFR) is a receptor protein tyrosine kinase that plays fundamental roles in normal and cancer cells [15]. Aberrant overexpression or overactivation of EGFR is associated with most types of epithelial cancers [16, 17]. In breast cancer, EGFR overexpression is associated with large tumor size, EMT, metastasis and poor clinical outcomes [18, 19]. EGFR is frequently overexpressed in triple-negative breast cancer (TNBC), a breast cancer type that is particularly aggressive and difficult to cure [20, 21]. Because of the crucial role of EGFR in breast cancer, several therapies that target EGFR, including gefitinib, cetuximab, lapatinib, and others, have been developed. However, results of clinical studies of EGFR-targeted therapy in breast cancer have been disappointing [22]. Thus, better understanding signaling mechanisms regulating EGFR expression and activation has become increasingly important for designing more effective therapeutic strategies.

MicroRNAs are a class of small (∼20–25 nucleotides), non-coding RNAs that bind to partially complementary target sites in messenger RNA (mRNA) 3′ untranslated regions (UTRs), which results in primarily blockage of translation and to some extent instability of the transcript [2]. Over 1000 microRNAs may be encoded by the human genome [23], and each of them is predicted to target tens to hundreds of different mRNAs [24] to regulate over 60% of human genes [25, 26]. Plenty of microRNAs have been reported to play roles in cancer [27]. In breast cancer, for example, the microRNA-200 family members have been shown to inhibit EMT and invasion in breast cancer [28].

In this study, we provide strong evidence that KLF8 promotes the expression of EGFR by both directly activating the gene promoter and by repressing its inhibitory microRNA141, a member of the microRNA-200 family to release its translation. This novel KLF8-miR141-EGFR signaling axis plays a potentially important role in breast cancer progression.

## RESULTS

### KLF8 and EGFR are highly co-overexpressed in human metastatic breast cancer patient tumors

**Figure 1:**
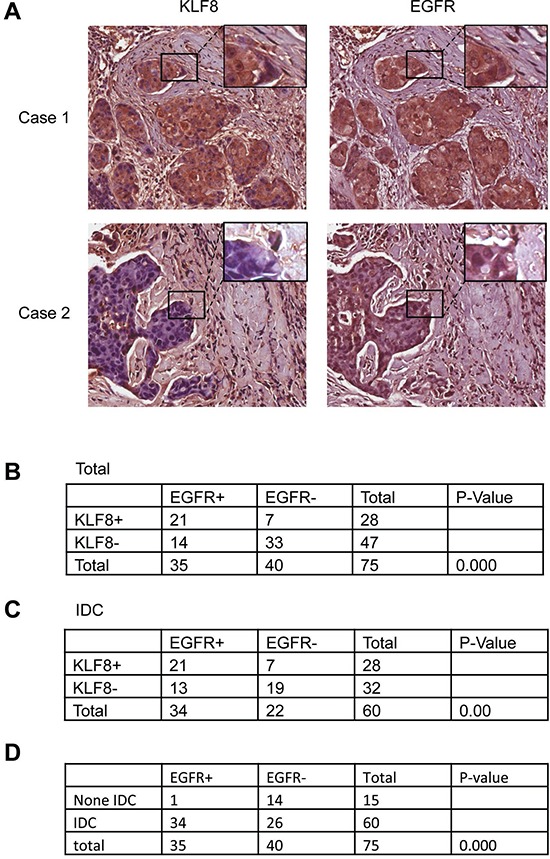
Correlated upregulation of KLF8 and EGFR expression in human breast cancer patient tumors **A.** IHC staining of EGFR or KLF8 (brown) in the human breast cancer tissue microarray was performed. Images representing specimens in duplicate from 75 patient tumors or normal tissues were shown. **B.** Correlation of EGFR and KLF8 expression was shown for all the samples. **C.** Correlation of EGFR and KLF8 expression was shown for all the samples in the invasive ductal carcinoma (IDC) specimens only. **D.** Positive correlation of EGFR expression with the invasive potential was outlined. Statistical significance was determined by χ^2^-test.

Our cDNA microarray data indicated that EGFR is potentially one of the highly KLF8-induced genes in the 10A-iK8 cells [29]. EGFR is overexpressed in TNBC cells such as MDA-MB-231 cells that also express aberrantly high level of KLF8 [7, 30]. We wanted to know whether the high expression of EGFR in the breast cancer cells or tissues [6, 7, 20] is associated with the high level of KLF8. We have recently performed a tissue microarray analysis of KLF8 expression in breast cancer patient tumors and have shown a positive correlation of KLF8 expression with invasive potential of the tumors [29]. To see whether EGFR expression in the same tissue microarray is correlated with KLF8 expression and the tumor invasive potential, we performed an immunohistochemical (IHC) staining for EGFR expression. Clearly, there was a higher level of EGFR expression in KLF8 positive, invasive tumors than that in KLF8 negative, non-invasive tumors (Figure 1A–1D). These results suggested that EGFR might be a KLF8-regulated target gene

### KLF8 upregulates EGFR expression at the transcriptional level

**Figure 2:**
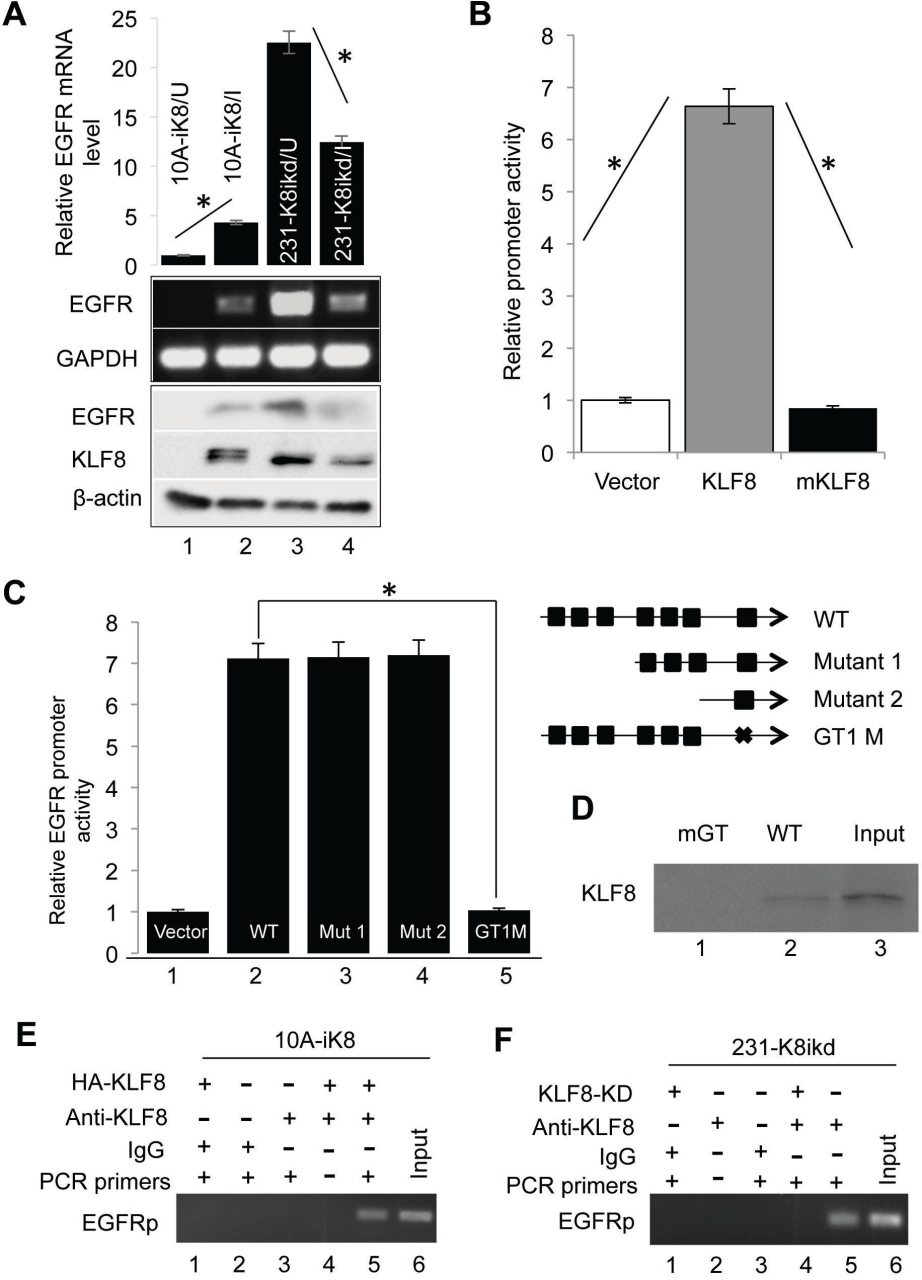
KLF8 upregulates EGFR expression at the transcriptional level **A.** KLF8 expression is both sufficient and necessary for EGFR expression. The 10A-ik8 and 231-k8ikd cells were grown under induced (I) or uninduced (U) conditions for 48 h. qPCR and western blotting were performed to determine the levels of EGFR in response to the change in KLF8 expression. **B.** KLF8 activates the human EGFR gene promoter (hEGFRp). The hEGFRp luciferase reporter plasmid was co-transfected into the NIH3T3 cells with wild-type KLF8, its activation domain-defective mutant (mKLF8) or the control vector plasmid for 16 h. The reporter activity was determined as described in the Material and Methods (*P < 0.01). **C.** The GT-box 1 (GT1) is required for the activation of hEGFRp by KLF8. The GT-boxes were individually mutated. The wild-type (WT) or mutant (mGT) reporters were used for similar reporter assay. *P < 0.01. **D.** KLF8 directly binds hEGFRp at the GT-box 1 site. HEK293 cell lysate containing HA-KLF8 and oligos spanning the wild-type GT1 (WT) or its mutant (mGT) were used for BOP assay as described in the Material and Methods. **E.** and **F.** Endogenous KLF8 binds hEGFRp *in vivo*. The 10A-iK8 or 231-K8ikd cells were cultured under the uninduced (U) or induced (I) conditions for 72 h. ChIP assays were done as described in the Material and Methods (see qPCR validation in Supplementary Figure 1A and 1B).

We further verified the high expression of EGFR [29] in the 10A-iK8 cells [31] when KLF8 overexpression was induced using qPCR and western blotting (Figure 2A, compare lanes 2 with 1). By contrast, EGFR expression was greatly decreased when KLF8 expression was knocked down in the 231-K8ikd cells [31] (Figure 2A, compare lanes 4 with 3).

To further investigate if EGFR is a transcriptional target of KLF8, we first searched the human EGFR promoter sequence in the region spanning the −2000 base pairs (hEGFRp) and found 7 GT boxes (CACCC or GGGTG, potential KLF8 binding sites). We then cloned the hEGFRp into pGL3basic luciferase reporter vector for promoter reporter assay. Co-expression of the wild type KLF8 caused a greater than 6-fold increase in the promoter activity whereas the activation domain-defective mutant of KLF8 [32] did not cause a change (Figure 2B). These data suggested that EGFR is potentially trans-activated by KLF8.

To determine if KLF8 directly binds the hEGFRp, we mutated the promoter to test which region responds to KLF8. We found that only when the GT-box 1 site was mutated, the hEGFRp was no longer activated by KLF8 (Figure 2C). This result suggested that the GT-box 1 is a strong candidate for KLF8 binding. Indeed, biotinylated oligonucleotide precipitation (BOP) (Figure 2D) and chromatin immunoprecipitation (ChIP) assays (Figure 2E & 2F and Supplementary Figure 1A & 1B) indicated that endogenous KLF8 could interact with the EGFR promoter at the GT-box 1 site and mutation of this site prevented the interaction (Figure 2D, lane 1).

Taken together, these results suggested that KLF8 binds to the human EGFR promoter at the GT-box 1 site to activate the transcription of EGFR.

### KLF8 represses miR141 to release EGFR

**Figure 3:**
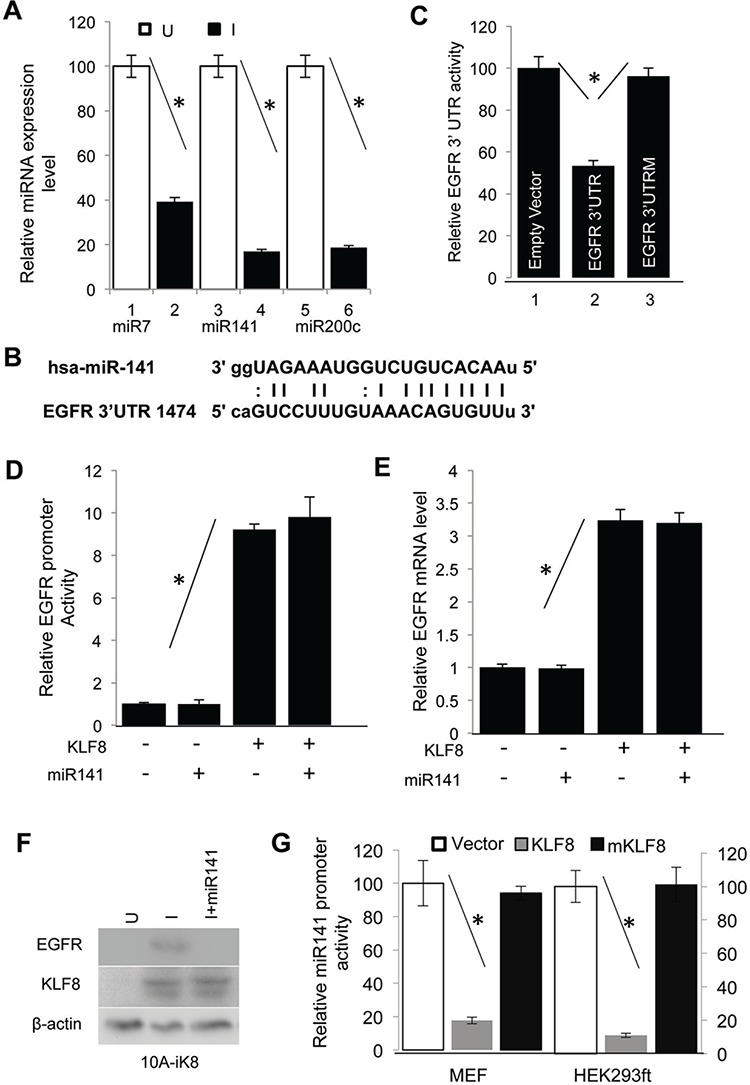
KLF8 upregulates EGFR protein levels by repressing miR141 **A.** KLF8 inhibits the expression of miR7, miR141 and miR200c. The 10A-iK8 cells were cultured under uninduced (U) or induced (I) conditions for 2 days. Then microRNAs were isolated. The expression of microRNAs was then detected by qPCR. **B.** The miR-141 binding site in the 3′-UTR of EGFR transcript was aligned with the miR-141 sequence. **C.** miR141 binds and represses the translation of EGFR transcript. The wild type (WT) 3′-UTR of EGFR or its mutant defective in miR-141 binding (EGFR 3′UTRM) luciferase reporter plasmid was co-transfected into the HEK293ft cells with the miR141 plasmid for reporter assays. **D.** miR141 expression does not affect the EGFR gene promoter. The 10A-iK8 cells with or without stable overexpression of miR141 were cultured with or without KLF8 induction for 2 days. Luciferase assay was then performed. **E.** miR141 expression does not affect EGFR expression at the mRNA level. The cells were treated similarly as described in 3c. qPCR was then followed. **F.** miR141 expression decreases EGFR expression at the protein levels. The cells were treated similarly as described in 3c. Cell lysate was then prepared for western blotting. **G.** KLF8 represses miR141 promoter. The human miR141 promoter luciferase reporter plasmid was co-transfected with KLF8, its repression motif-defective mutant (mKLF8) or empty vector. Luciferase reporter assay was followed (see qRT-PCR validation in Supplementary Figure 1C and 1D).

We noticed a significantly larger effect of KLF8 on EGFR protein levels than its promoter activity (Figure 2). This indicated that some other mechanisms might also exist downstream of KLF8 to promote EGFR expression at the post-transcriptional levels such as translation of the transcript and stabilization of the protein. MicroRNAs are known to bind to the 3′-UTR of the gene transcript to primarily block the translation. Interestingly, by searching candidate microRNAs that bind to the human EGFR 3′-UTR using TargetScan 5.1, miRanda, miRbase and PITA software programs and our recent microRNA expression profiling analysis [6], we came up with 2 microRNAs, miR7 and miR141, that were predicted to be able to bind to the human EGFR 3′-UTR (Figure 3A) and that both of the microRNAs are downregulated in KLF8-overepressed cells [33]. miR7 has been reported to repress EGFR in glioblastoma [34]. miR141 belongs to the microRNA-200 family that is known to inhibit EMT and metastasis [35] by inhibiting the EMT-inducing ZEB1 and SIP1 [28, 36]. To our best knowledge, there has been no report on regulation of EGFR by miR141 yet. To test the potential role of miR7 and miR141 on EGFR expression in our experimental system, we first confirmed the microRNA array data by qPCR (Figure 3A). We then cloned the 3′-UTR cDNA of human EGFR transcript containing the matched site for miR141 (Figure 3B) into the pIS0 luciferase reporter plasmid [33, 37]. Co-transfection of miR141 and the reporter plasmid suggested a dramatic repression in the 3′-UTR (Figure 3C, lane 2). By contrast, this repression was not seen if the miR141 matched site was mutated (Figure 3C, lane 3). Overexpression of miR141 did not affect EGFR promoter activity (Figure 3D) and mRNA expression (Figure 3E) induced by KLF8, but abolished the protein level of EGFR induced by KLF8 (Figure 3F). This result suggested that miR141 binds the matched site and inhibits the translation of EGFR [2]. We next tested if miR141 is a direct repressive target of KLF8. We cloned the promoter of miR141 into the luciferase reporter plasmid and performed the promoter reporter assay. KLF8 repressed the promoter activity by approximately 80% whereas a repression domain-defective mutant of KLF8 (mKLF8) [5, 8, 32, 38] could not (Figure 3G). This result was further supported by the repression of miR141 expression by KLF8 in these cells similarly as described above (Supplementary Figure 1C & 1D and Figure 3A). These data suggested that miR141 is a likely direct repression target of KLF8.

Taken together, our results strongly suggested that in addition to directly activating the EGFR gene promoter, KLF8 also represses miR141 to promote the translation of EGFR.

### KLF8 promotes invasion and proliferation depending upon EGFR

**Figure 4:**
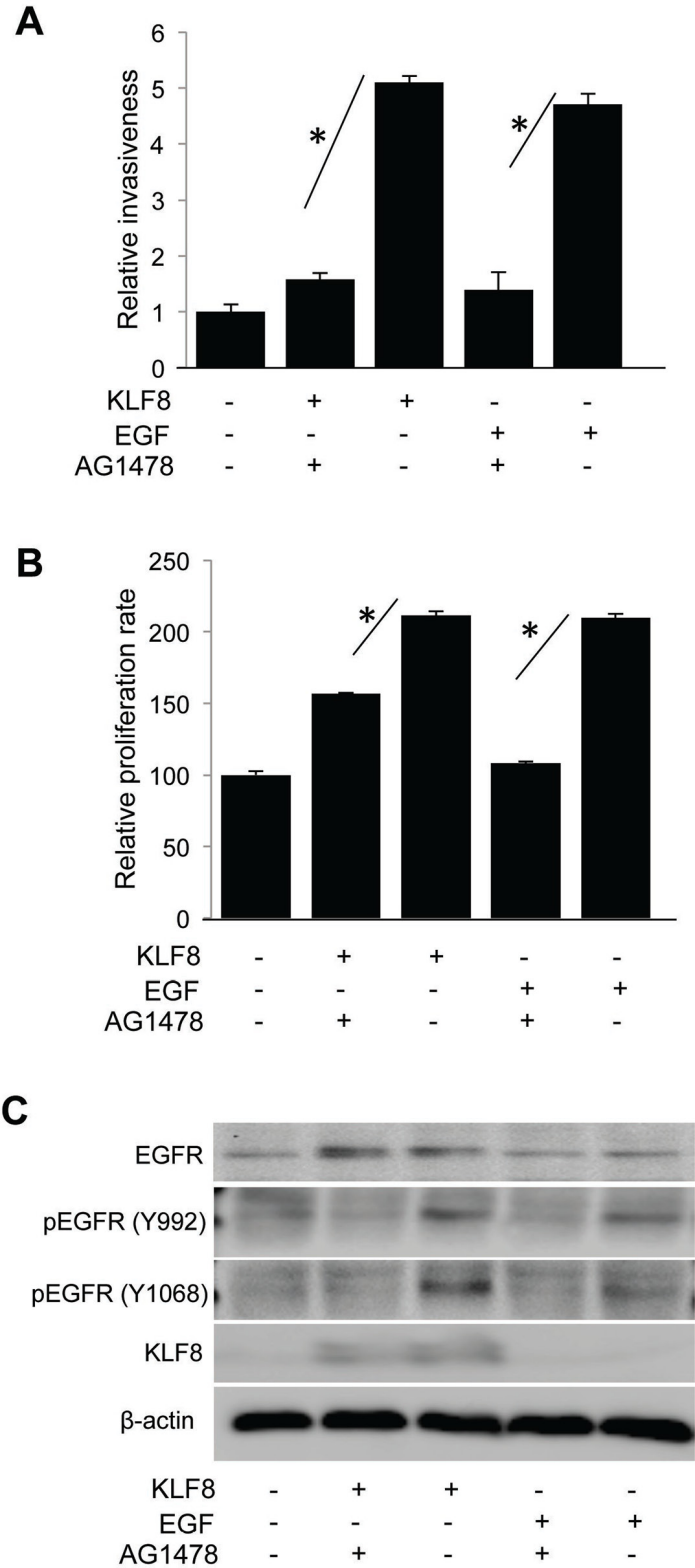
KLF8 promotes EGFR-dependent invasion and proliferation The 10A-iK8 cells were cultured under uninduced (U) or induced (I) conditions for 2 days. Then the cells were treated with DMSO or AG1478 for 30 min and prepared for Matrigel invasion assay **A.** or WST-1 assay **B.** as described in the Materials and Methods. Inhibition of EGFR by AG1478 was verified by western blotting **C.** EGF treatment was included as a control. *P < 0.05.

We have demonstrated that KLF8 promotes EMT, migration, invasion and metastasis by repressing E-cadherin and activating MMP2, MMP9, and MMP14 [2, 6, 7]. EGFR is well known to promote breast cancer progression [16, 18, 21, 30, 39, 40]. We tested whether EGFR regulates these functions downstream of KLF8. We found out that inhibition of EGFR activity using AG1478 could reduce invasiveness (Figure 4A) and proliferation (Figure 4B) in the 10A-iK8 cells induced by KLF8 overexpression or EGF stimulation. Western blotting results showed that EGFR inhibitor decreases EGFR phosphorylation at Y992 and Y1068 irrespective of KLF8 overexpression (Figure 4C). These results suggest that the expression and activation of EGFR are critical downstream of KLF8 for the cell invasion and proliferation.

### MiR141 counteracts KLF8 to inhibit invasion and proliferation

**Figure 5:**
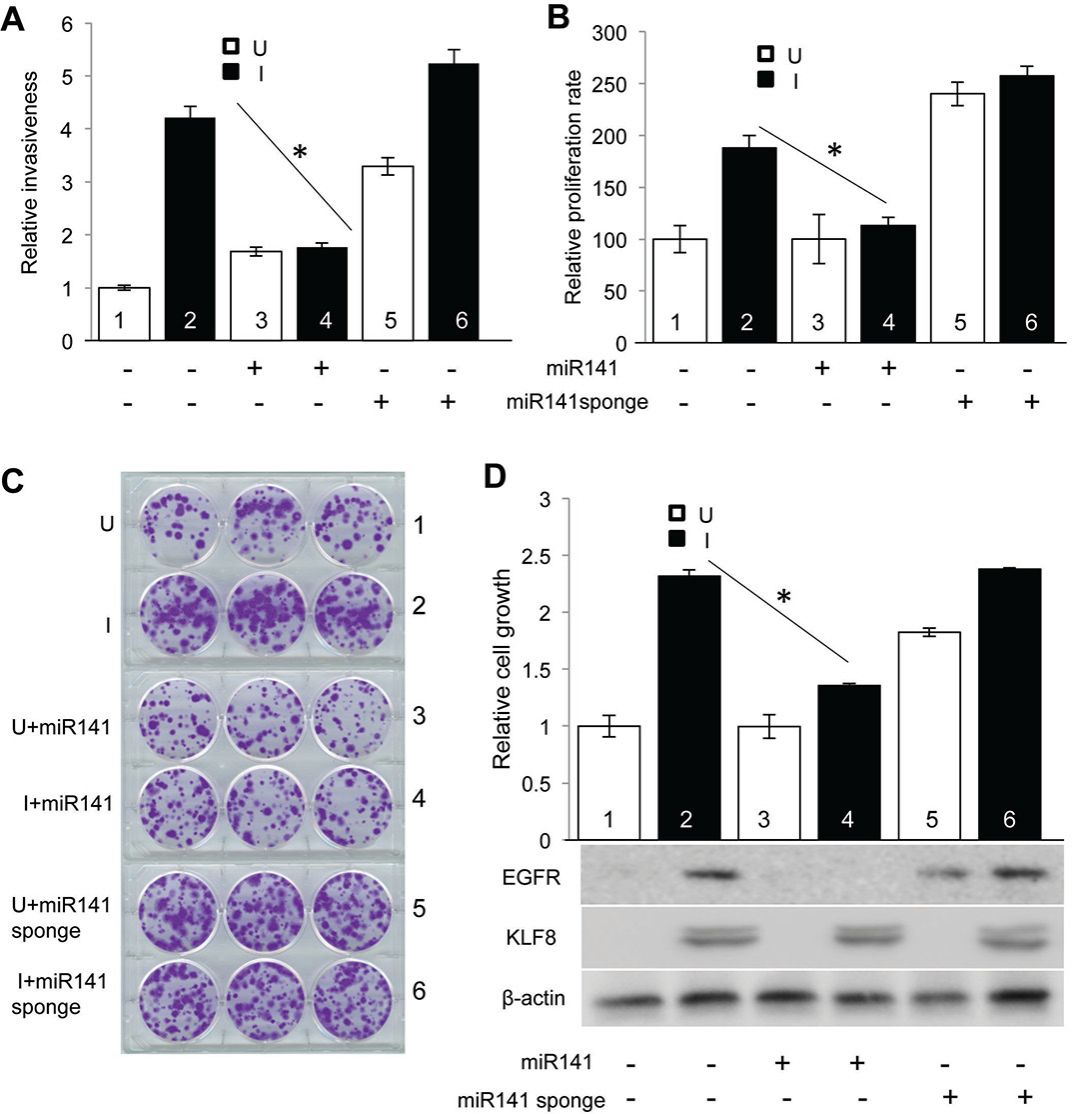
Downregulation of miR141 is required for KLF8 to promote invasion and proliferation **A.** Overexpression of miR141 blocks KLF8-promoted invasion. The 10A-iK8, 10A-iK8-miR141, and 10A-iK8 miR141sponge cells were cultured under uninduced (U) or induced (I) conditions for 2 days. Then Matrigel invasion assay was performed. Serum was used as the chemoattractant. **B.** Overexpression of miR141 blocks KLF8-promoted proliferation. The cells were treated as described in 5A. Then WST-1 assay was performed as described in Materials and Methods. **C.** and **D.** Overexpression of miR141 blocks KLF8-promoted cell viability. The cells were treated as described in panel 5A. Then clonogenic assay was performed as described in Materials and Methods. The pictures of the colonies were shown in panel 5C. After imaged, the cells were washed with methanol and the OD 540 value was measured by colorimetry. Western blotting was performed using cell lysate prepared from parallel plates for KLF8 and EGFR as shown in panel 5D.

To test the impact of miR141 downstream of KLF8, we made two stable cell lines derived from 10A-iK8, one with miR141 overexpression (10A-iK8-miR141) and the other with miR141 sponge [6] overexpression (10A-iK8-miR141 sponge). We found that miR141 overexpression blocked the KLF8-induced the cell invasiveness in Matrigel (Figure 5A), proliferation by WST- assay (Figure 5B) and viability by clonogenic assay (Figure 5C) (compare lanes 2 and 4). By contrast, inhibition of miR141 by the sponge promotes these cellular functions to an extent comparable with that by KLF8 overexpression (compare lanes 5 with 2). However, combination of miR141 sponge and KLF8 overexpression did not further enhance these roles (compare lanes 6 with 2 or 5). Western blotting (Figure 5D) results showed that miR141 repressed EGFR expression induced by KLF8 and inhibition of miR141 upregulated EGFR even in the absence of KLF8 overexpression. These results indicate that KLF8 repression of miR141 and subsequent activation of EGFR are critical for the cell invasion and growth.

### KLF8 promotes proliferation via the EGFR-ERK axis

**Figure 6:**
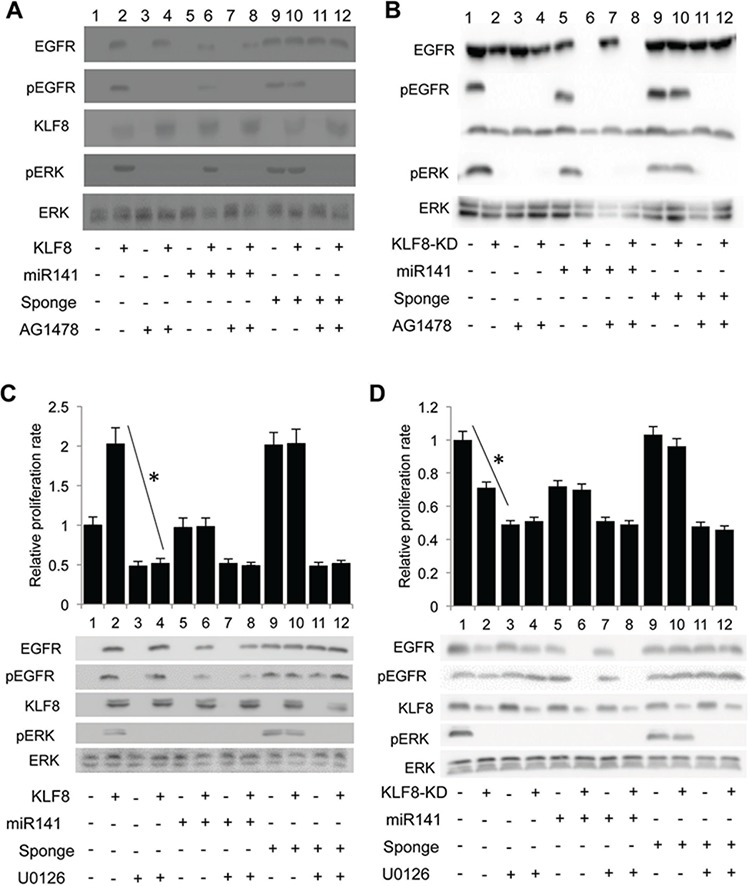
The KLF8-promoted proliferation is mediated by the EGFR-ERK axis **A.** and **B.** Inhibition of EGFR blocks the KLF8-dependent activation of ERK. The 10A-iK8, 10A-iK8-miR141, and 10A-iK8 miR141sponge cells (A) or the 231-K8ikd, 231-K8ikd-miR141, and 231-K8ikd-miR141sponge cells (B) were cultured under uninduced (U) or induced (I) conditions for 2 (A) or 3 (B) days. DMSO or AG1478 was then added into the medium for 30 min. After changing fresh medium replacement, the cells were cultured for one additional hour and then cell lysates were prepared for western blotting. **C.** and **D.** Inhibition of MEK abolishes the KLF8-dependent proliferation. The same cell sets and culture conditions as described in panels 6A and 6B were followed. Then DMSO or the MEK inhibitor U0126 was added into the medium for 15 min. The cells were then reseeded, incubated for 24 hours followed by WST-1 assay and western blotting. *P < 0.01.

ERK is a major mediator of EGFR signaling [41, 42] as well as KLF8 signaling [9]. Our results showed that in the 10A-iK8 cells [31]KLF8 promoted the phosphorylation of EGFR and ERK1/2 (pEGFR, pERK) (Figure 6A, compare lanes 2 and 1) that was prevented by the EGFR inhibitor (Figure 6A, compare lanes 4 and 3). Overexpression of miR141 decreased pEGFR and pERK induced by KLF8 (Figure 6A, compare lanes 6 and 2). Inhibition of miR141 increased the activation of EGFR and ERK even in the absence of KLF8 overexpression (Figure 6A, compare lanes 9 and 1). We also made two cell lines derived from the 231-K8ikd cells [31], one with overexpression of miR141 (231-K8ikd-miR141) and the other with overexpression of miR141 sponge (231-K8ikd-miR141sponge). In these cells, knocking-down of KLF8 decreased the levels of pEGFR and pERK (Figure 6B. compare lanes 2 and 1). Overexpression of miR141 decreased the levels of pEGFR and pERK (Figure 6B. compare lanes 5 and 1). Inhibition of miR141 by the sponge increased the levels of pEGFR and pERK activation (Figure 6B. compare lanes 2 and 10). We further used MEK inhibitor U0126 [43] to inhibit the pathway even downstream of EGFR. The results showed that the MEK inhibitor dramatically decreased cell proliferation induced by KLF8 or miR141 sponge (Figure 6C and 6D). Western blotting results showed that the MEK inhibitor totally blocked ERK activation but not EGFR activation in the cells.

Taken together, these data suggest that KLF8 promotes proliferation primarily via the miR141-EGFR-ERK axis in the cells.

### Knockdown of KLF8 or overexpression of miR141 inhibits tumor growth and invasion

**Figure 7:**
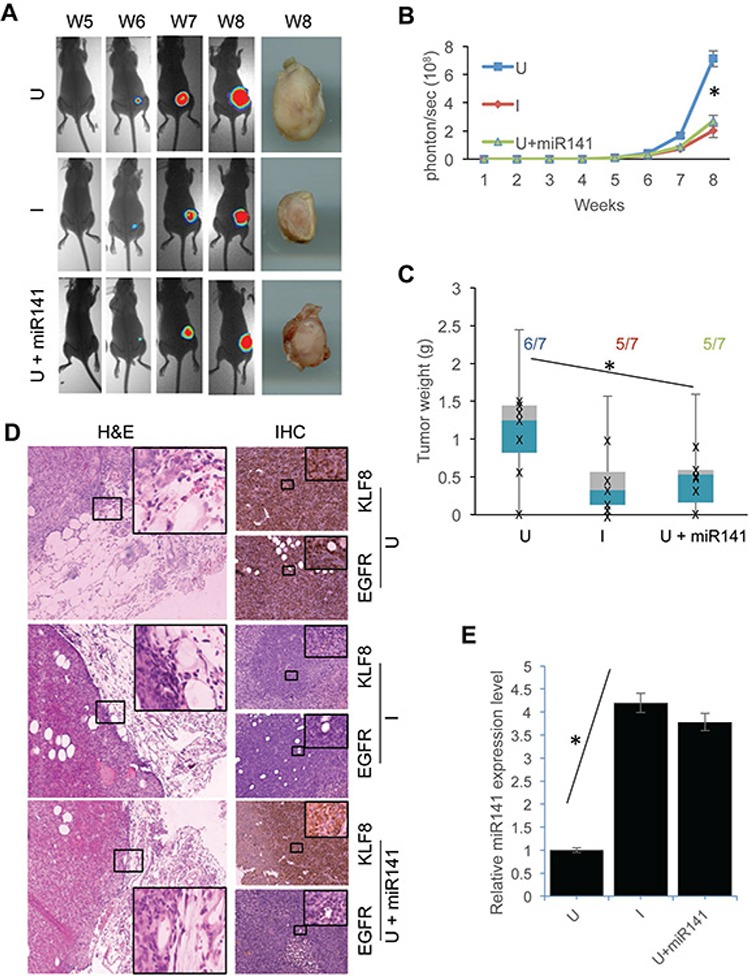
Overexpression of miR141 recapitulates the inhibitory effect of KLF8 knockdown on the tumor growth and invasion in the breasts The 231-K8ikd cells or 231-K8ikd-miR141 cells (U + miR141) were injected into the mammary fat pad. The mice injected with 231-K8ikd cells were fed with food supplemented with (I, KLF8 knockdown) or without doxycycline (U, no KLF8 knockdown). The mice injected with the 231-K8ikd-miR141 cells were fed with normal control food. The tumor growth was followed up for 8 weeks by BLI or stereomicroscopy at week 8 as described in Materials and Methods. **A.** Representative BLI and tumor images. **B.** and **C.** Quantitative results of the time course BLI intensity of tumor growth or tumor weights at week 8. The numbers indicate the tumor formation incidence. **D.** Knockdown of KLF8 or overexpression of miR141 inhibits the tumor invasion and decreases EGFR protein levels in the tumor tissues. The tumor samples prepared on week 8 were processed for H&E staining for tumor invasion (with basement membranes or tumor-adjacent interfaces highlighted) and IHC staining for the expression of KLF8 and EGFR proteins. **E.** Knockdown of KLF8 causes an increase in miR141 expression in the tumor tissues. microRNAs were prepared from the same tumors as described in panel d for qPCR analysis of the expression of miR141. *P < 0.05.

To determine to what extent the KLF8-miR141-EGFR axis affects tumor progression in the breast, we orthotopically injected the 231-K8ikd cells or the 231-K8ikd-miR141 cells into the mammary fat pad and then measured the tumor growth and invasion (Figure 7). We found that the tumor growth was inhibited comparably by the knockdown of KLF8 and the overexpression of miR141 (U + miR141) (Figure 7A–7C). Importantly, tumor invasion into the surrounding tissue was also inhibited in the same experimental conditions (Figure 7D). IHC staining indicated that KLF8 and EGFR were correlated in expression (Figure 7A, U & I) and overexpression of miR141 decreased EGFR expression without affecting KLF8 expression in the tumors (Figure 7D, U + miR141). qPCR analysis confirmed the overexpression of miR141 in the 231-K8ikd-miR141 cells as well as the upregulation of the endogenous miR141 upon KLF8 knockdown in the tumor tissue (Figure 7E). These results confirmed the *in vitro* results described above and suggest that the regulation of proliferation, survival and invasiveness by the KLF8-miR141-EGFR signaling axis is a critical mechanism responsible for the tumor growth and invasion *in vivo*.

### Knockdown of KLF8 or overexpression of miR141 inhibits tumor metastasis to the lungs

**Figure 8:**
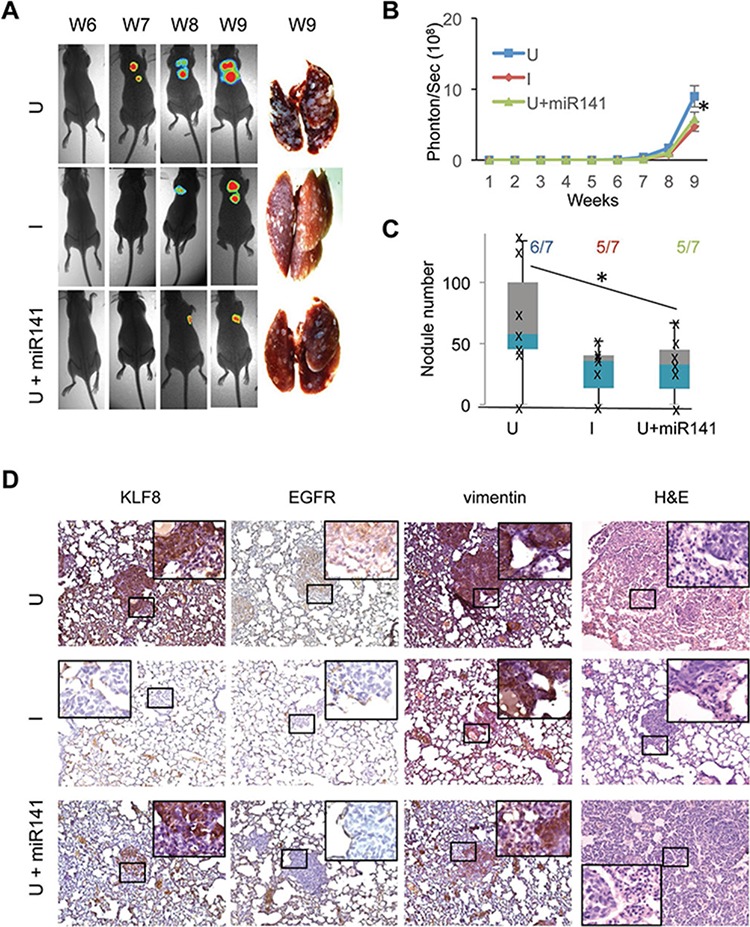
Overexpression of miR141 recapitulates the inhibitory effect of KLF8 knockdown on the tumor metastasis to the lungs The 231-K8ikd or 231-K8ikd-miR141 cells were injected into the tail vein. The mice were fed as described in Figure 7. BLI analysis and stereomicroscopy of the tumors **A.** and **B.** and H&E and IHC staining analyses **D.** were performed similarly as in Figure 7. Anti-human vimentin staining was included to confirm the human origin of the lung metastases. **C.** The number of tumor nodules on the surface of the lungs as well as the lung metastasis incidence were shown. *P < 0.05.

We have recently showed that KLF8 promotes breast cancer lung metastasis [2, 6]. To test whether the miR141-EGFR axis plays a mediating role, we injected the 231-K8ikd cells or the 231-K8iKD-miR141 cells into the tail vein and then measured the lung metastasis by bioluminescent imaging (BLI) (Figure 8A and 8B). Lung metastasis began to be detected by BLI at week 6–7 and became eye-visible on the lungs at week 9 (Figure 8A–8C). KLF8 knockdown (I) and miR141 overexpression (U + miR141) both significantly slowed the metastasis rate (Figure 8A and 8B) and the number of metastasis nodules (Figure 8C) compared with uninduced group (U). The human origin of tumors was verified by human-specific vimentin IHC staining (Figure 8D). IHC staining using KLF8 and EGFR antibody indicated the co-expression of the two proteins and this correlation was abolished by miR141 expression (Figure 8D). These results suggest that the KLF8-miR141-EGFR signaling axis plays a critical role for the lung metastasis of breast cancer.

## DISCUSSION

This report identified a novel KLF8 to EGFR signaling mechanism for the tumor growth and lung metastasis of human breast cancer. In this signaling model of tumor progression, the abnormally high expression of KLF8 in the cancer cells ensures the high level of EGFR. On the one hand, KLF8 activates EGFR at the transcriptional level by directly binding to the gene promoter. On the other, KLF8 represses miR141 expression to release EGFR from translation repression by the microRNA, resulting in a further increase in the protein levels of EGFR. The high levels of EGFR protein and the downstream pathway including ERK in the cells warrant the subsequent tumor growth, invasion and metastasis.

EGFR plays a crucial role in the breast tumor growth and metastasis [39]. Our cDNA microarray analysis identified EGFR as one of the most upregulated genes by KLF8 and we have validated this result at both mRNA and protein levels (Figure 2). Furthermore, we have correlated the co-expression of KLF8 and EGFR with the metastatic potential of the patient tumors (Figure 1). In addition, KLF8 and EGFR share some common target genes, including cyclin D1 [9, 44], MMP9 [6, 45, 46], and promotes similar cellular events such as EMT, proliferation and metastasis [2, 6, 11, 47, 48]. Altogether, these results strongly suggest that EGFR may be another KLF8 target important in tumor progression of breast cancer patients.

Our data are consistent with a recent report that the expression of miR141 and EGFR is inversely correlated in breast cancer [49]. However, to the best of our knowledge, this is the first report showing that miR141 binds the EGFR transcript and represses its translation. miR141 is known to function as a tumor suppressor by repressing ZEB1, one of the strong EMT inducers [28, 35, 36]. miR141 belongs to the miR-200 family and this family of microRNAs plays a crucial role in inhibiting EMT, invasion and metastasis [30, 31]. We have identified both miR141 and miR200c as targets of repression by KLF8 by microRNA microarray analysis [6]. Since miR141 and miR200c share the same promoter and KLF8 strongly represses this promoter (Figure 3G), it is likely that the regulation of miR200c and ZEB1 by KLF8 also contributes to the overall outcomes of the tumor progression. This will be an interesting future topic of research. We also identified miR7 as a target of repression by KLF8 [6] (Figure 3). miR7 has been reported to inhibit EGFR [34]. Although modestly repressed by KLF8, miR7 may also play a partial role in the regulation of EGFR expression downstream of KLF8, which is worth of further investigation.

EGFR-targeted clinical trials have not yielded convincing outcomes for breast cancer [22]. This novel KLF8-miR141-EGFR signaling axis presented here could be explored for designing alternative therapeutic strategies. In summary, this work provides significant new insights into the mechanisms of breast cancer progression and opens a new gate in the study of KLF8, miR141 and EGFR in breast cancer as potential biomarkers or therapeutic targets.

## MATERIALS AND METHODS

### Cell culture and reagents

#### Antibodies

Antibodies used for western blotting were EGF receptor rabbit polyclonal antibody (#4267), Phospho-EGF receptor (Tyr992) rabbit polyclonal Ab (#2235), Phospho-EGF receptor (Tyr1068) rabbit polyclonal Ab (#3777) (Cell Signaling Technology, Inc., Danvers, MA, USA), β-actin (C4) mouse monoclonal Ab (sc-47778), p-ERK (E-4) mouse monoclonal Ab (sc-7383), ERK 1 (C-16) rabbit polyclonal Ab (sc-93), HA-probe (F-7) mouse monoclonal Ab (sc-7392), (Santa Cruz Biotechnology, Inc., Dallas, Texas, USA), human vimentin mouse monoclonal Ab (550513) (BD Biosciences Inc, San Jose, CA, USA), HRP-conjugated donkey anti-mouse IgG (715-035-150) or anti-rabbit IgG (711-035-152), Peroxidase substrate kit (DAB) (SK-4100) (Vector laboratories Inc., Burlingame, CA, USA). Antibodies used for BOP and ChIP assays were HA-probe (F-7) and KLF8 rabbit polyclonal Ab (8477), respectively as previously reported [7, 8, 11].

#### Plasmids

pLenti 4.1 Ex miR200c-141 was purchased from Addgene (#35534. Cambridge, MA, USA). The human miR141 cDNA was cloned and inserted into pLenti 4.1 between the EcoR I and Xho I sites to generate pLenti 4.1-miR141 plasmid. The 3′-UTR of human EGFR cDNA (hEGFR-3′-UTR) was cloned and inserted into pIS0 vector [37] (#12178. Addgene. Cambridge, MA, USA) between the Sac I and Xba I sites to generate pIS0-hEGFR3′UTR plasmid. The promoter of human EGFR gene (hEGFRp) was cloned and inserted into PGL3b vector between the Kpn I and Nar I sites to generate pGL3b-hEGFRp plasmid. The miR141 sponge was inserted into the pBABE- puro-mCherry vector between the EcoR I and Age I sites to generate pBABE-puro-mcherry-miR141sponge plasmid. The human miR141 promoter (hmiR141p) was cloned and inserted into pGL3b between the Nhe I and Hind III sites to generate pGL3b-hmiR141p plasmid.

#### Cell lines

The HEK293 and NIH3T3 [50–52], MCF-10A, and MDA-MB-231 [9, 14], and the KLF8-expressing Tet-on MCF-10A (10A-iK8) and the KLF8 shRNA-expressing Tet-on MDA-MB-231 (231-K8ikd) cell lines were described elsewhere [31]. These cells were maintained in DMEM/F-12 or DMEM with 10% fetal bovine serum or calf serum. The 10A-iK8-miR141 and 231-K8ikd-miR141 were generated by infecting 10A-iK8 and 231-K8ikd cells with viruses derived from pLenti 4.1 miR141 followed by puromycin selection. The 10A-iK8-miR141sponge and 231-K8ikd-miR141sponge cell lines were generated by infecting the cells with viruses derived from the pBABE-puro-mcherry-miR141sponge plasmid followed by puromycin selection. The cell lines were maintained under U (in the absence of doxycycline) or I (in the presence of doxycycline) conditions depending on the experimental need. Doxycycline hydrochloride was purchased from Sigma-Aldrich Corp. (D3072, St. Louis, MO, USA).

### Quantitative real-time PCR (qPCR), western blotting, promoter reporter assays, chromatin immunoprecipitation (ChIP) and biotinylated oligonucleotide precipitation (BOP)

These assays were performed as previously described [8, 9, 32]. The plasmids encoding the wild-type or mutant hEGFRp, hmiR141p or human EGFR 3′-UTR reporter and the control vector, HEK293, MEF, 10A-iK8 and 231-K8ikd cell lines were described above.

### Bioluminescence imaging (BLI) analysis of tumor growth and metastasis

This analysis was done similarly as reported earlier [4, 53]. All animal work was done in accordance with a protocol approved by the Institutional Animal Care and Use Committee. Female athymic Nude-Foxn1nu nude mice (NCI) of 4 - 6 weeks old were used. An amount of 10^6^ viable 231-K8ikd or 231-K8-ikd-miR141 cells were washed and harvested in 0.1 ml phosphate-buffered saline and subsequently injected into the tail vein or mammary fat pad of the mice. The mice were fed with diet supplemented with doxycycline (Dox Diet, S3888. Bio Servs, Frenchtown, NJ, USA) to induce the knockdown of KLF8 expression in the cells *in vivo* or with the Control Diet not containing doxycycline (S4207). After injection, tumor growth or lung metastasis was monitored daily or weekly visually and/or by BLI. For BLI, mice were anaesthetized and injected with an i.p. dose of 150 mg/kg of D-luciferin (15 mg/ml in PBS) (LUCK-1, Gold Biotechnology, Inc., St. Louis, MO, USA). Imaging was completed in 3 min after injection with the Kodak Carestream Imaging System coupled to analysis software.

### Matrigel invasion assay

Matrigel invasion assays were done as described previously [7, 31, 53] using BD BioCoat invasion chambers and serum in the complete medium as the chemoattractant. Except for the presence of the Matrigel, the invasion chambers were incubated with the culture medium for 2 hours at 37°C. Then 5 × 10^4^ cells were loaded into the top chamber. After 18 hour incubation, the chamber was scratched and washed following by crystal violet staining.

### WST-1 assay

This proliferation assay was performed essentially as reported previously [53, 54]. Briefly, 2000 cells were seeded into 96 wells plates. After 24 hour incubation, the WST-1 substrate was added into the medium and the cells were then cultured for additional two hours prior to quantification of the absorbance of each sample using a microplate reader at a wavelength of 450 nm.

### Clonogenic assay

This cell viability assay was carried out as reported earlier [53, 54]. Briefly, 2000 cells were seeded into each well of 6-well plates. After incubated for 14 days, the cells were stained with crystal violet and photographed. The cells were then washed with methanol for colorimetric quantification under OD 540.

### Hematoxylin and eosin (H&E) and immunohistochemical (IHC) staining

The collection and processes of mammary tissues and the lungs, the human breast cancer tissue array, and the staining procedures were previously described [4, 31, 53, 55]. The antibodies specific for KLF8, EGFR and human vimentin were described above. Analysis of metastatic tumor nodules on the surface of the lungs was carried out as previously described [53].

### Statistical analysis

Summary data are presented as mean + standard deviation with a minimum of three observations per group. Unpaired, paired or single sample Student's *t*-test with the Bonferroni correction for the multiple comparisons was applied as appropriate. The two by two tables for human data were analyzed by Fisher's exact test. Significance was determined by the alpha level of 0.05.

## SUPPLEMENTARY FIGURE


