# Supplementary material for: Identification of epidermal growth factor receptor and its inhibitory microRNA141 as novel targets of Krüppel-like factor 8 in breast cancer
Source: Oncotarget. 2015 May 27;6(25):21428–42. doi: 10.18632/oncotarget.4077 (PMC4673276; doi:10.18632/oncotarget.4077)
Supplement: Supplementary file 1 [file oncotarget-06-21428-s001.pdf]

SUPPLEMENTARY FIGURE

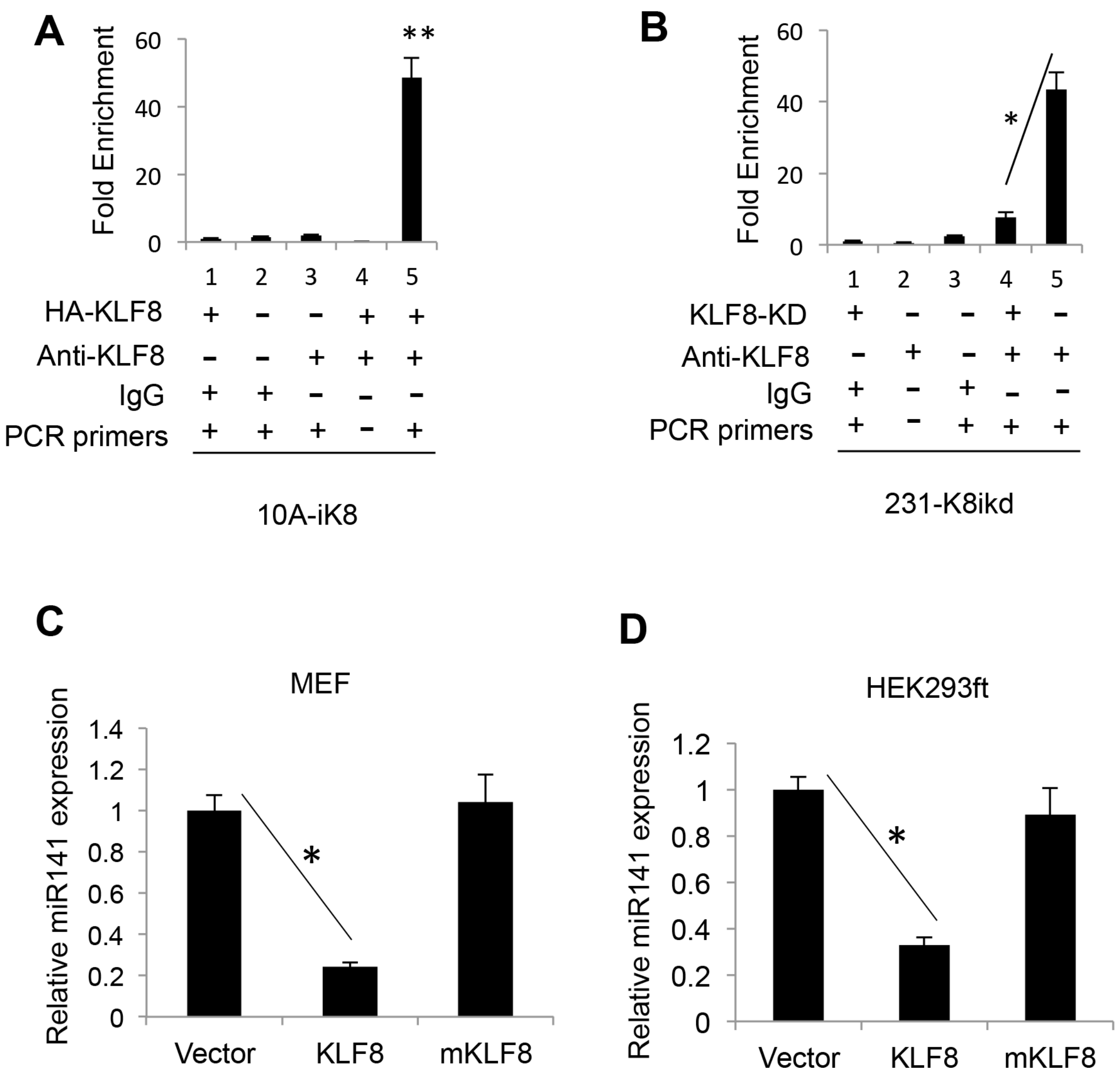

Supplementary Figure 1: A. & B. Supplements to Figure 2E & 2F. The ChIP assay results were validated by quantitative PCR. The relative folds to IgG control were compared. **C. & D.** Supplements to Figure 3G. The promoter reporter assay results were validated by qRT-PCR analysis of changes in miR-141 in expression by KLF8. **P* < 0.05; ***P* < 0.01.
